# Discovery of Sesamolin as a Potential Anti-*Helicobacter pylori* Agent by Virtual Screening against the Essential Response Regulator HsrA with *In Vitro* Evaluation

**DOI:** 10.1021/acsomega.5c08531

**Published:** 2025-11-11

**Authors:** Yihang Xu, Shuhan Luo, Fen Yang, Baochao Zhang, Xiaoqin He, Juan Liao, Lili Liao, Tianli Zheng, Xiaofang Pei

**Affiliations:** West China School of Public Health and West China Fourth Hospital, 12530 Sichuan University, Chengdu 610041, China

## Abstract

*Helicobacter pylori* infects nearly half of the world’s population and contributes to chronic gastritis, gastric cancer, and other gastrointestinal diseases. Upon diagnosis, antibiotic-based regimens are typically used for eradication. However, the increasing antibiotic resistance of *H. pylori* and the side effects of these medications highlight the need for safer and more effective alternatives, particularly those derived from natural products. In this study, by using high-throughput molecular docking, 12 commercially available potential HsrA inhibitors were screened out from 13,142 natural compounds in the TCMSP database for the first time. After evaluation of their binding-mode stability to HsrA by using molecular dynamics (MD) simulations and their minimum inhibitory concentration (MIC), sesamolin (**3**) and coptisine (**6**), exhibiting stable binding modes and strong anti-*H. pylori* activity, were selected, and their inhibitory effects on the DNA-binding activity of HsrA were further confirmed using electrophoretic mobility shift assay (EMSA). However, compound **3** bound HsrA with high affinity (*K*_D_ = 17.08 nM), approximately 23-fold stronger than compound **6** (*K*_D_ = 399.60 nM). Further RT-qPCR analysis demonstrated that compound **3** exerted regulatory effects stronger than those of **6** on HsrA-linked genes (*tlpB*, *flhA*), urease genes (*ureA*, *ureB*), and the oxidative stress gene (*sodB*; reported to inversely correlate with HsrA levels). Consistently, **3** showed a greater suppression of urea chemotaxis and a larger increase in superoxide dismutase (SOD) activity than **6**. Our investigation uncovered sesamolin, a common lignan found in sesame, which could be a potential anti-*H. pylori* compound by binding with HsrA and regulating the related key genes of *H. pylori*.

## Graphical abstract

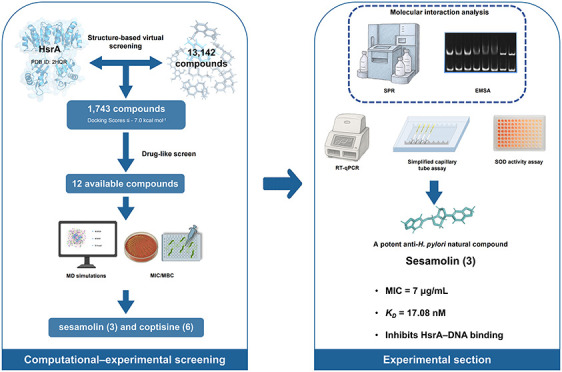

## 1 Introduction

*Helicobacter pylori*, a microaerophilic Gram-negative bacterium, is transmitted *via* person-to-person contact, zoonotic exposure, and ingestion of contaminated food or water.^1^ It has a high global infection rate of approximately 43.9% among adults and 35.1% among children and adolescents^2^ and contributes to gastrointestinal diseases like peptic ulcers and gastric cancer, having been classified as a Group 1 carcinogen by the IARC in 1994.^1,3–5^ Moreover, emerging evidence implicated that *H. pylori* infection was related to neurological disorders such as Alzheimer’s disease, multiple sclerosis, and Parkinson’s disease.^6^ Therefore, the strategy for managing *H. pylori* infection for all individuals is to provide eradication therapy.

The initial eradication therapy typically includes either a bismuth-based quadruple regimen, which consists of a proton-pump inhibitor (PPI), bismuth, and two antibiotics such as metronidazole (MTZ) plus tetracycline or amoxicillin plus clarithromycin, or rifabutin-based triple therapy, which includes a PPI, rifabutin, and amoxicillin.^4,5,7,8^ However, the poor patient compliance due to medication side effects and long treatment durations, as well as the rising antibiotic resistance of *H. pylori*, have contributed to the global decline in eradication rates.^9,10^ Therefore, the search for safer and more effective alternatives, particularly those derived from natural products to prevent *H. pylori* infection, is of paramount importance, and a number of studies have focused on screening various plant extracts, traditional Chinese medicines, and natural products.^11,12^ For example, we screened out edible rose (*Rosa rugosa* cv. “Plena”) as a potential antibacterial agent against multidrug-resistant *H. pylori* from 17 plants, and other studies demonstrated that natural products such as flavonoids, terpenoids, and alkaloids present in the plants and herbal medicines exhibited inhibitory effects against *H. pylori* by using traditional culturing methods.^11–14^ However, these approaches are often time-consuming and resource-intensive. Accordingly, rapid and cost-effective virtual screening (VS) has attracted growing interest.^15^ It has been applied for screening anti-*H. pylori* compounds from StreptomeDB,^16^ Chemdiv,^17^ and ZINC^18^ database by targeting DAH7PS, *secA*, and HsrA, respectively. Nevertheless, VS does not guarantee the exclusion of inactive compounds, and further postscreen refinements (*e.g.*, MM-GBSA, MM-PBSA, and molecular dynamics simulations) as well as experimental validation are usually required.^19–21^

HsrA (encoded by *hp1043*) is an essential transcription factor of *H. pylori*, featuring an N-terminal dimerization domain and a C-terminal DNA-binding domain (DBD).^22^ It acts by binding multiple promoters to control vital processes such as transcription, translation, and energy metabolism, and its conservation and multifunctionality make it a promising therapeutic target for *H. pylori* infection.^23^ González et al. screened out seven HsrA inhibitors from 1120 small molecules in a commercial chemical library by using fluorescence-based thermal shift assay, opening the possibility of a drug-repurposing approach for the treatment of *H. pylori* infection.^22^

High-throughput virtual screening (HTVS) is a computational approach in drug discovery that efficiently identifies potential bioactive molecules from large libraries by simulating molecular interactions using molecular docking and molecular dynamics (MD) simulations, thereby reducing experimental workload and cost compared to traditional experimental screening.^24^ Antoniciello et al. screened three HsrA inhibitors from 14,350 compounds in the ZINC database by using HTVS, including molecular docking and MD simulations. In light of these encouraging findings, we applied a similar strategy with modifications to screen 13,142 natural compounds against HsrA from the Traditional Chinese Medicine Systems Pharmacology (TCMSP) database for the first time, since traditional Chinese medicine provides an important anti-*H. pylori* sources with remarkable curative effect, high safety, less drug resistance, and improvement of clinical symptoms.^7,25^ TCMSP database serves as a comprehensive pharmacology platform that integrates extensive information, including pharmacochemical data, ADME characteristics of Chinese herbal medicine, *etc.*

In our study, we first applied Autodock Vina to perform high-throughput molecular docking with HsrA. Commercially available natural compounds with high docking scores were selected, followed by preliminary 50 ns MD simulations to investigate their molecular motions and interactions over time. Since HsrA plays a crucial regulatory role in *H. pylori*, binding of natural compounds to HsrA may interfere with its function and inhibit bacterial growth. Thereby, anti-*H. pylori* activities of the natural compounds were subsequently evaluated by using minimum inhibitory concentration (MIC) testing against an MTZ-resistant strain. Furthermore, the binding sites, stability, and free energy of natural compounds with HsrA were further assessed using extended 200 ns MD simulations. These compounds were selected based on their stable binding in preliminary simulations or excellent antibacterial activity *in vitro*. Subsequently, electrophoretic mobility shift assays (EMSA), which track changes in protein–DNA complex mobility, and surface plasmon resonance (SPR), a label-free, real-time biosensing technique, were employed to confirm the binding of the selected compounds to HsrA. Given that consensus HsrA-DNA-binding motifs are present in the promoter regions of *tlpB* and *flhA*, which are important for *H. pylori* colonization and motility—with *tlpB* involved in environmental sensing^26^ and *flhA* in flagellar assembly^27^—and that HsrA is proposed to act as a repressor of *sodB*,^28^ we applied RT-qPCR to confirm the influence of selected compounds after binding with HsrA on the expression of these three genes, together with the influence on other core functional genes (*sodB*, *ureA*, *ureB*, and *secA*). Besides, time-kill kinetics, chemotaxis, and SOD assays were also conducted to further evaluate the effects of selected compounds on the growth, the urea chemotaxis ability, and the SOD activity of *H. pylori*, thereby to reveal their activity as potential HsrA inhibitors.^28^

## 2 Results

### 2.1 Structure-Based Virtual Screening

In this study, a total of 13,142 natural compounds retrieved from this database were screened against HsrA using Autodock Vina, and their docking scores were obtained. Among them, 1743 natural compounds had the docking scores below the threshold (−7.0 kcal mol^–1^)^29^ and fulfilled the physicochemical and pharmacokinetic property requirements of Lipinski’s rule, the Ghose filter rule, and Veber’s rule; these are summarized in Table S1 and listed in ascending order according to their docking scores. Subsequently, 12 commercially available, relatively inexpensive natural compounds with the lowest docking scores were selected for further testing. The information on these 12 compounds, including molecular structure, formula, natural source, and docking scores (in ascending order), is shown in Table 1.

**Table 1:** Docking Scores of Twelve Natural Compounds against HsrA

compound	docking score (kcal mol^–1^)
Mulberroside C (**1**)	–9.7
Limonin (**2**)	–9.2
Sesamolin (**3**)	–9.2
Tanshinone IIA (**4**)	–9.1
Glycitin (**5**)	–9.1
Coptisine (**6**)	–9.1
Berlambine (**7**)	–8.9
Calycosin-7-*O*-β-d-Glucopyranoside (**8**)	–8.9
Emodin (**9**)	–8.8
Obacunone (**10**)	–8.8
Indirubin (**11**)	–8.7
Baicalein (**12**)	–8.7

### 2.2 Pocket Identification and Conservation Grades

To predict potential binding sites of these 12 natural compounds on HsrA, the DoGSiteScorer server was employed, and five potential binding pockets were observed. These pockets varied in volume from 543.84 to 1089.39 Å^3^ and surface area from 793.3 to 1645.22 Å^2^. The simple scores ranged between 0.32 and 0.64, while the druggability scores were all relatively high, ranging from 0.798 to 0.845 on a scale from 0 to 1, indicating favorable druggable sites. Specifically, Pocket 1 exhibited the largest volume (1089.39 Å^3^) and a druggability score of 0.818, whereas Pocket 3 had the highest druggability score of 0.845 despite a smaller volume (768.06 Å^3^). The detailed information is demonstrated in Table S2. Furthermore, the ConSurf server was used to map the evolutionary conservation grades of HsrA, and the results are visualized in Figure 1A. The predicted binding pockets largely overlap with regions of high evolutionary conservation. Notably, pockets 1, 2, 3, and 5 correspond to the DBD, while pocket 4 suggests a distinct binding mode. The highest conservation scores (9, dark red) are concentrated within residues 118–216 of the DBD, consistent with previous studies.^30^ Besides, we also used the PLIP online tool to analyze the interactions of the selected natural compounds with HsrA, including H-bond forming, hydrophobic interactions, salt-bridges, and π-alkyl forces. The binding energies and protein–ligand interaction profiles of these 12 natural compounds with HsrA are presented in Table S3, sorted in ascending order of binding energy. Mapping PLIP contacts onto DoGSite cavities indicated that most ligands interact with pockets associated with the DBD: compounds **3**, **4**, **8**, and **9** primarily bound Pocket 1; compounds **2** and **6** bound Pocket 5; and compounds **7** and **11** bound Pockets 1 and 3. By contrast, compound **12** bound Pocket 4 exclusively, whereas compounds **1**, **5**, and **10** also sampled Pocket 4 (Table S4).

**Figure 1:**
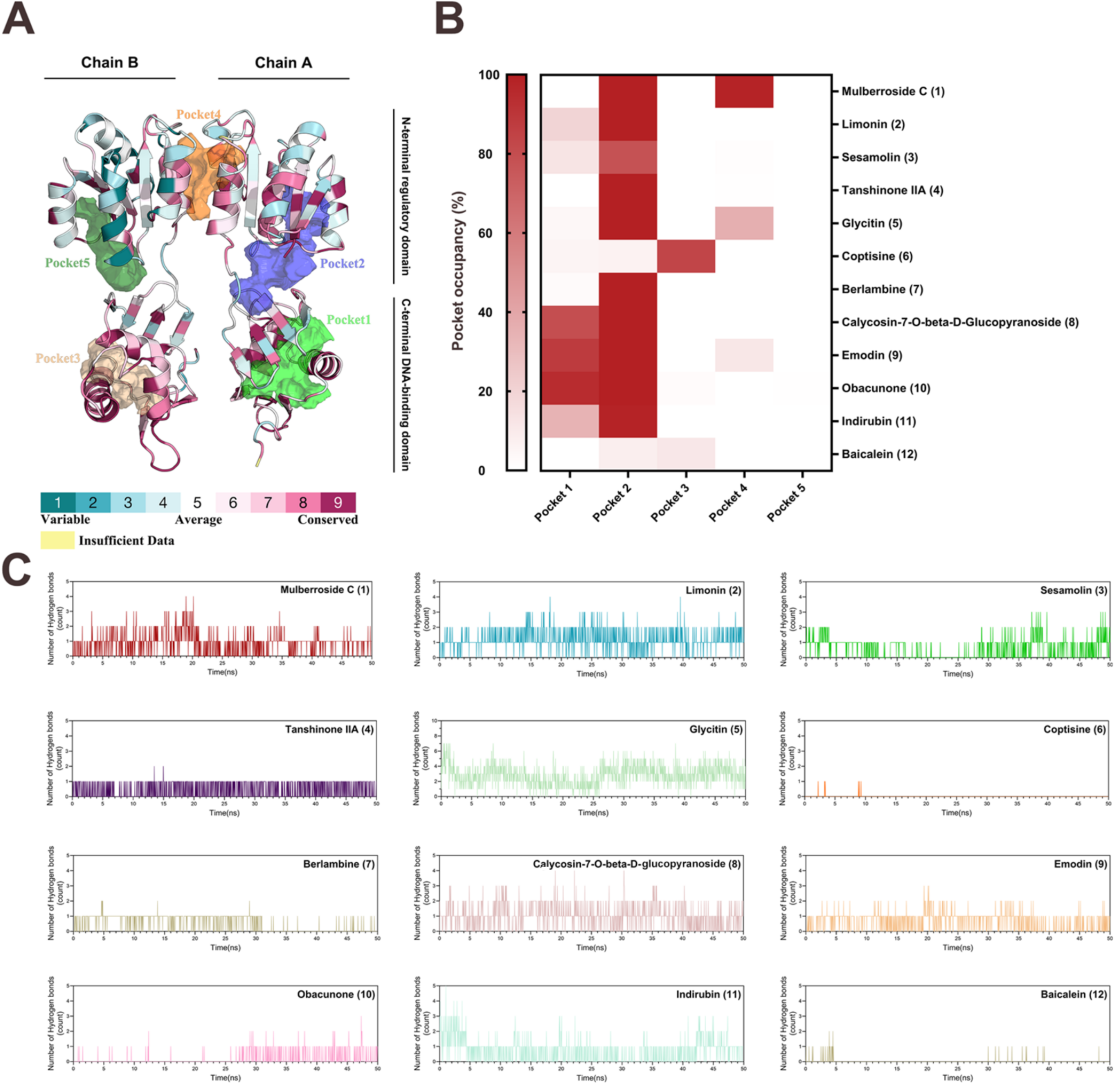
HsrA structural conservation and binding dynamics with natural compounds. (A) Evolutionary conservation of HsrA (cyan, low; maroon, high) and predicted drug-binding site (transparent colored surface) mapped onto the HsrA dimer (PDB ID: 2HQR). (B) Pocket occupancy heatmap from 50 ns MD: fraction of frames with any ligand heavy atom within 4.0 Å of DoGSiteScorer pocket residues. (C) Number of H-bonds between ligands and HsrA over time during 50 ns MD simulations.

### 2.3 Preliminary Molecular Dynamics (MD) Simulation

In this study, we initially performed 50 ns molecular dynamics (MD) simulations using GROMACS to assess the binding stability of the selected natural compounds and their effects on the protein residues. Root-mean-square deviation (RMSD) and fluctuation (RMSF) values of the protein–ligand complexes were calculated to assess their dynamic behaviors, with RMSD reflecting complex stability and RMSF indicating protein residue flexibility. RMSD profiles for the complex, protein backbone, and ligand of all 12 compounds are presented in Figure S2. The backbone and ligand RMSD traces indicated that the HsrA complexes with compounds **2**, **6**, **7**, and **11** reached an apparent plateau after initial relaxation. Compound 3 showed partial stabilization with a gradual upward drift, whereas compounds **1**, **4**, **5**, **8**, **9**, **10**, and **12** did not converge within 50 ns, displaying persistent upward trends or large fluctuations. In the 50 ns MD simulations, ligand motions related to the protein cavities are summarized in Figure 1B and Table S5. When ligand–protein contact frames were mapped onto the cavities, most ligands preferentially occupied DBD Pocket 2, while Pocket 1 was sampled only sporadically. Contacts with pockets 3, 4, and 5 were rare and typically transient, except for compound **6**, which favored pocket 3. The number of H-bonds formed between HsrA and the natural compounds in the simulation period is also shown in Figure 1C and Table 2. Notably, no H-bonds were observed for compound **6**. As shown in Figure S3, for all 12 complexes studied, chain A-B center-of-mass (COM), N-terminal domain–domain (NTD-NTD), and DNA-binding domain–domain (DBD-DBD) distances remained near steady levels with only small fluctuations, indicating no separation of chains A and B.

**Table 2:** Molecular Dynamics (MD) Metrics of Twelve Natural Compounds

compound	RMSD (Å) (mean ± SD)	RMSF (Å) (mean ± SD)	H-bonds^a^
Mulberroside C (**1**)	9.47 ± 0.93	3.11 ± 1.14	0.87
Limonin (**2**)	6.90 ± 0.44	2.44 ± 0.87	1.17
Sesamolin (**3**)	6.84 ± 0.75	2.86 ± 0.92	0.57
Tanshinone IIA (**4**)	7.11 ± 1.10	2.41 ± 0.84	0.43
Glycitin (**5**)	7.55 ± 0.93	3.07 ± 1.36	2.78
Coptisine (**6**)	6.32 ± 0.30	2.39 ± 0.97	0.00
Berlambine (**7**)	5.71 ± 0.37	1.45 ± 0.83	0.52
Calycosin-7-*O*-β-d-Glucopyranoside (**8**)	6.65 ± 0.60	2.60 ± 1.09	1.00
Emodin (**9**)	5.67 ± 0.62	2.98 ± 1.13	0.65
Obacunone (**10**)	7.08 ± 0.68	2.39 ± 0.84	0.15
Indirubin (**11**)	6.24 ± 0.48	2.24 ± 0.76	0.69
Baicalein (**12**)	7.68 ± 0.75	2.98 ± 1.09	0.02

a Average number of hydrogen bonds in preliminary MD simulations (50 ns).

### 2.4 Inhibitory Effects of 12 Natural Compounds on *H. pylori*

The MICs for 12 natural compounds against ATCC 43504 *H. pylori* strains were evaluated using the microbroth dilution method. As shown in Table 3, compounds **3**, **6**, **9**, and **10** exhibited relatively low MIC values (7, 16, 64, and 64 μg/mL), and their minimum bactericidal concentration (MBC) was subsequently tested. The results are also demonstrated in Table 3, and we found that compounds **3** and **6** had the strongest anti-*H. pylori* effects, and their binding interactions with HsrA were further analyzed by using extended MD simulations.

**Table 3:** MIC and MBC of Twelve Natural Compounds against MTZ-Resistant *H. pylori* (ATCC 43504)

compound	MIC (μg/mL)	MIC (μM)	MBC (μg/mL)	MBC (μM)
Sesamolin (**3**)	7	18.9	10	27.0
Coptisine (**6**)	16	49.9	32	99.9
Emodin (**9**)	64	236.8	64	236.8
Obacunone (**10**)	64	140.8	128	281.6
Mulberroside C (**1**)	128	279.2	n.d.	n.d.
Limonin (**2**)	256	544.1	n.d.	n.d.
Tanshinone IIA (**4**)	256	869.9	n.d.	n.d.
Glycitin (**5**)	>1024	>2293.9	n.d.	n.d.
Berlambine (**7**)	>1024	>2914.1	n.d.	n.d.
Calycosin-7-O-β-d-Glucopyranoside (**8**)	>1024	>2293.9	n.d.	n.d.
Indirubin (**11**)	>1024	>2991.5	n.d.	n.d.
Baicalein (**12**)	>1024	>3789.2	n.d.	n.d.

### 2.5 Extended Molecular Dynamics Simulation

Extended MD simulations were performed with 200 ns for compounds **3** and **6**. As shown in Figure 2A, the RMSD analysis of compound **3** exhibited rapid system equilibration reaching convergence by 10 ns, followed by a secondary structural rearrangement between 50 and 100 ns, and maintained restrained fluctuations around a dynamic equilibrium value of 0.893 ± 0.042 nm until the end of the 200 ns simulation. In contrast, compound **6** reached convergence at 130 ns and maintained restrained fluctuations around a dynamic equilibrium value of 0.760 ± 0.031 nm until the end of simulation. Compound **3** demonstrated faster equilibration and more stable backbone conformations, which implied better conformational stability than **6**. Compound **3** occupies pockets 1 and 2, while **6** occupies pocket 2 (Table S5). As shown in Figure 2B, compound **3** formed an average of 0.57 H-bonds, whereas compound **6** formed only 0.03.

**Figure 2:**
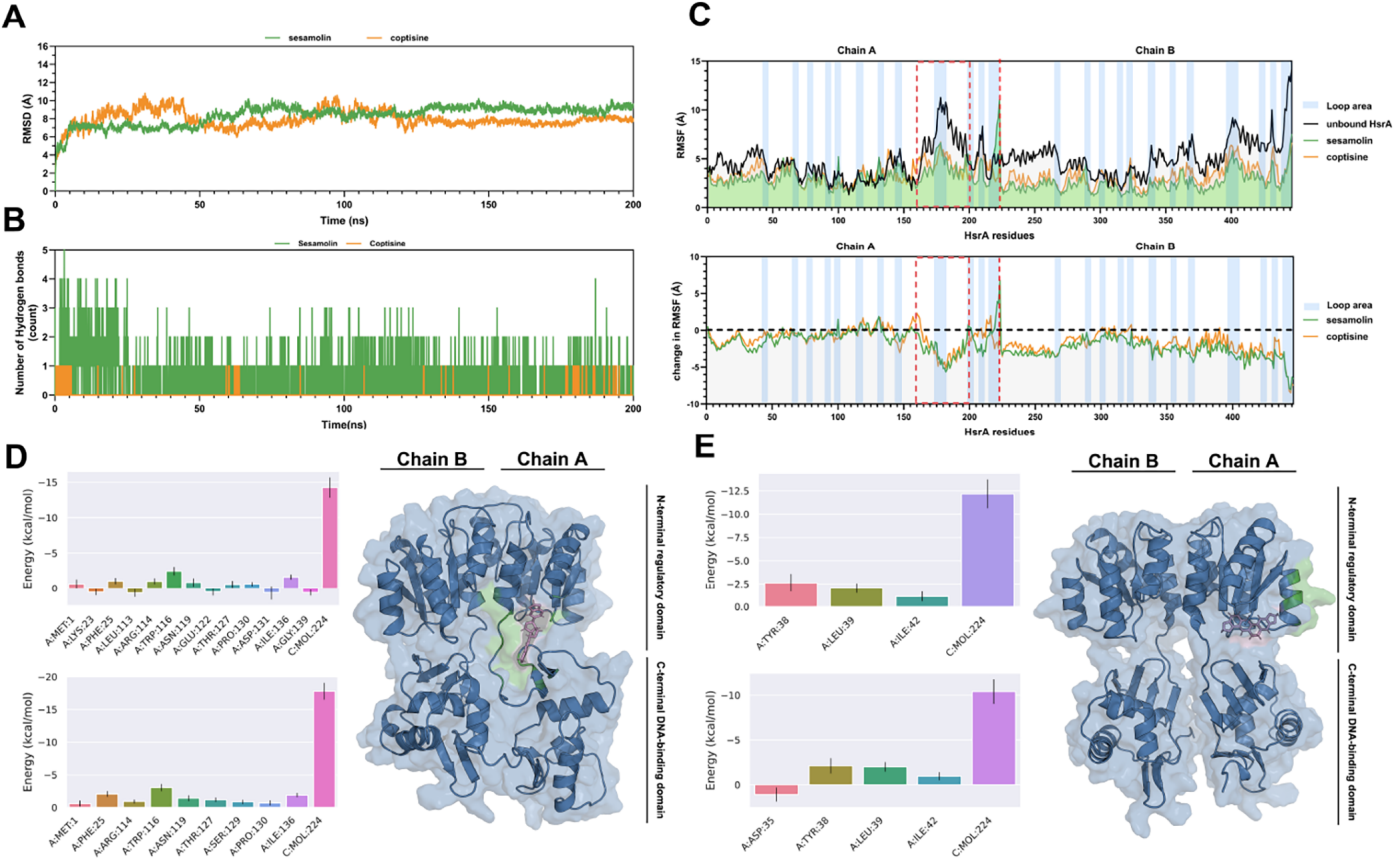
Binding mechanism analysis of HsrA with sesamolin (**3**) and coptisine (**6**). (A) Protein RMSD (compound **3** in green, compound **6** in orange). (B) HsrA-ligand H-bonds (compound **3** in green, compound **6** in orange). (C) Protein RMSF and protein change in RMSF (unbound HsrA in black, **3**-bound HsrA in green, **6**-bound HsrA in orange). (D) Compound **3**: Energy decomposition (GB/PB; residue threshold: 0.01 kcal mol^–1^) and pose with stabilizing residues (Δ*G* < −0.5 kcal mol^–1^, green). (E) Compound **6**: Energy decomposition (GB/PB; residue threshold: 0.01 kcal mol^–1^) and pose with stabilizing residues (Δ*G* < −0.5 kcal mol^–1^, green).

Furthermore, RMSF was used to evaluate the flexibility of HsrA as well as its complexes with compounds **3** and **6**. As shown in Figure 2C, characteristic peaks of unbound HsrA were observed in flexible loop domains, especially at residues 160–200, which were inside the DNADBD (highlighted in the red box). After the reaction with compounds **3** and **6**, decreased RMSF peaks were noticed, with the most pronounced reduction observed in the 160–200 residue region. Notably, among the 446 residues of HsrA, 412 residues exhibited decreased RMSF values upon binding with compound **3**, with an average reduction of 41.7 ± 16.4%, while for compound **6**, the reduction was 32.9 ± 15.4%, indicating that HsrA became more stable when bound to compound **3**.

The binding free energies, including the total binding free energies (Δ*G*_total) and calculated binding free energies (Δ*G*_binding), of compounds **3** and **6** with HsrA, were evaluated by using both the MM-GBSA (GB) and MM-PBSA (PB) models. As shown in Table 4, for compound **3**, Δ*G*_total obtained from the GB and PB models were −30.62 ± 2.39 kcal mol^–1^ and −24.92 ± 2.85 kcal mol^–1^, while those for compound **6** were −18.00 ± 2.18 kcal mol^–1^ and −14.44 ± 2.33 kcal mol^–1^. After entropy correction using the Interaction Entropy (IE) method, compound **3** exhibited Δ*G*_binding values of −23.96 ±  2.39 kcal mol^–1^ and −18.26 ±  2.85 kcal mol^–1^ for the GB and PB models, respectively, while the corresponding values for compound **6** were −11.81  ±  2.19 and −8.25 ±  2.34 kcal mol^–1^. These results indicate that compound **3** exhibits a notably stronger binding affinity toward HsrA compared to compound **6**. Between GB and PB energies for both compounds, van der Waals interactions (Δ*E*_vdW) were the primary contributors to binding. The Δ*E*_vdW of compounds **3** and **6** were found to be −44.13 ± 2.40 and −21.52 ± 2.25 kcal mol^–1^, respectively. Whereas electrostatic interactions (Δ*E*_EL) provided a secondary contributor to the binding of compound **3**, its Δ*E*_EL was −8.33 ± 3.02 kcal mol^–1^, but electrostatic interactions did not contribute to the binding of compound **6**.

**Table 4:** Binding Free Energy Decomposition Analysis Using MM-GBSA and MM-PBSA Models^a^

	HsrA_sesamolin (3)		HsrA_coptisine (6)	
energy component	GB	PB	GB	PB
ΔGGAS	–52.46 ± 3.39	–52.46 ± 3.39	–21.52 ± 2.25	–21.52 ± 2.25
ΔEvdW	–44.13 ± 2.40	–44.13 ± 2.40	–21.52 ± 2.25	–21.52 ± 2.25
ΔEEL	–8.33 ± 3.02	–8.33 ± 3.02	0.00	0.00
ΔGSOLV	21.84 ± 0.53	27.54 ± 3.11	3.52 ± 0.03	7.08 ± 1.52
ΔEGB/ΔEPB^b^	27.08 ± 2.18	31.62 ± 3.12	6.15 ± 0.93	6.73 ± 1.58
ΔESURF/ΔENPOLAR	–5.24 ± 0.16	–4.08 ± 0.09	–2.64 ± 0.29	0.35 ± 0.16
Δ*G*_total	–30.62 ± 2.39	–24.92 ± 2.85	–18.00 ± 2.18	–14.44 ± 2.33
Δ*G*_binding^c^	–23.96 ± 2.39	–18.26 ± 2.85	–11.81 ± 2.19	–8.25 ± 2.34

a GB, generalized Born; PB, Poisson–Boltzmann. Values in kcal mol^–1^.

b ΔEGB, generalized Born solvation energy in GB model; ΔEPB: polar solvation energy in PB model.

c Entropy-corrected *via* Interaction Entropy method.

Binding free energy decomposition results demonstrated that compound **3** preferentially targets the C-terminus, which was the DNA-binding site of HsrA (residues 118–216) and the linking domain (residues 113–117) (Figure 2D), suggesting its functionality through direct interaction with pockets 2 or 5. In comparison, compound **6**, with fewer residues contributing to binding, primarily interacts with regions near the N-terminus (Figure 2E).

### 2.6 Comparison of Effects of Compounds **3** and **6** on Anti-*H. pylori*

To further compare their anti-*H. pylori* activities, time-kill kinetic assays were conducted using the metronidazole-resistant *H. pylori* strain ATCC 43504. Both compounds were tested at the same set of concentrations, with 100 μM metronidazole (MTZ) and BHI broth serving as the resistance and negative controls, respectively. As shown in Figure 3, ATCC 43504 maintained robust growth under high-dose MTZ exposure, similar to the negative control (without the anti-*H. pylori* agent). Notably, compound **3** exhibited potent bactericidal activity, completely killing *H. pylori* within 12 h at 40 μM and within 8 h at 60 and 80 μM. Even at 20 μM, it effectively suppressed this bacterial growth within 48 h. In contrast, compound **6** showed weaker anti-*H. pylori* activity, achieving bacterial killing only at 80 μM within 12 h and inhibiting growth at 60 μM after 48 h of exposure.

**Figure 3:**
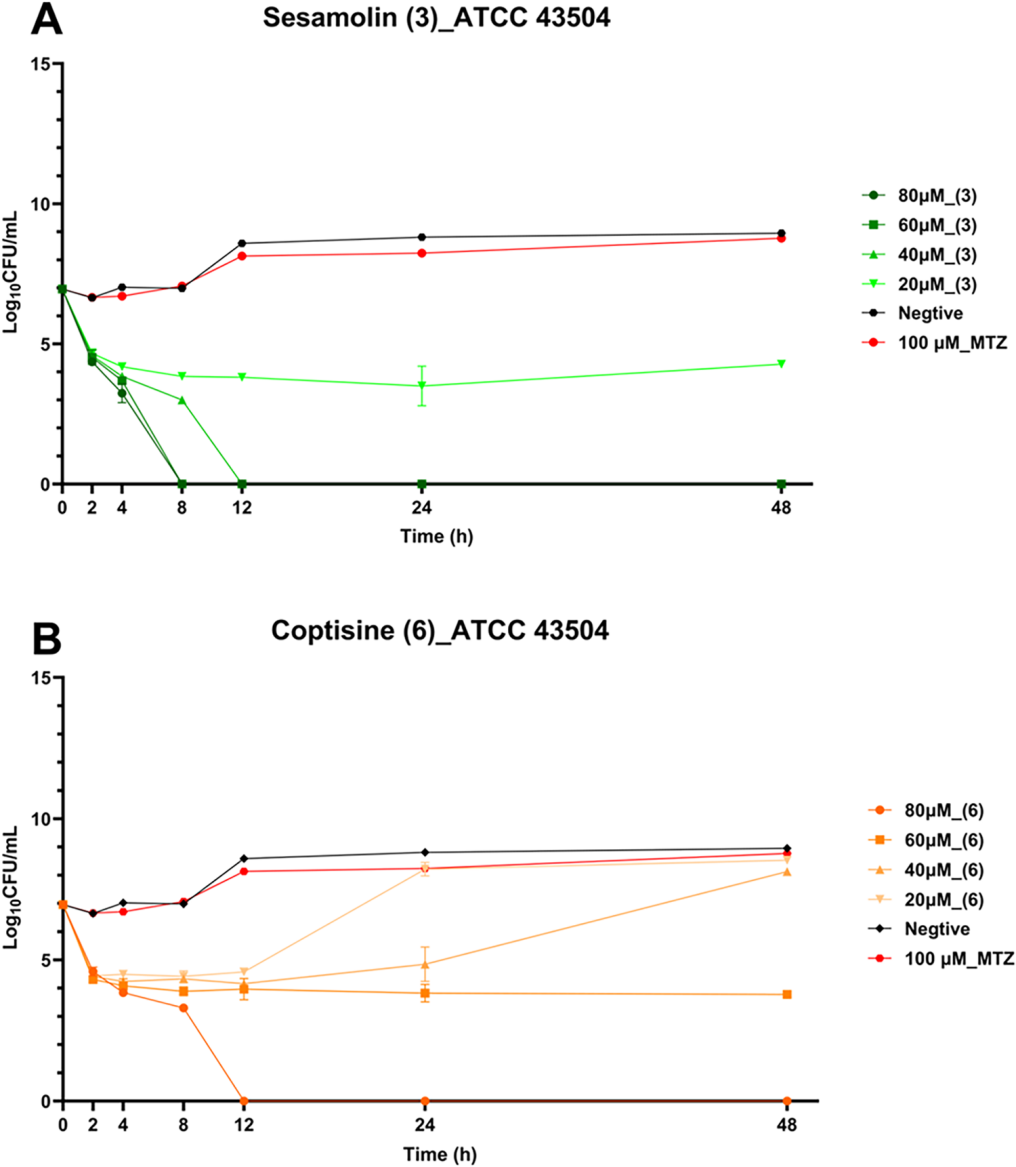
Time-kill kinetics of (A) sesamolin (**3**) and (B) coptisine (**6**) against *H. pylori* strain ATCC 43504. The experiment was repeated three times, but the error lines were too small and were obscured by the marked points in the figure.

### 2.7 EMSA and SPR Evaluation of HsrA Inhibition by Compounds 3 and 6

To further assess the inhibitory effects of compounds **3** and **6** on HsrA, we performed *in vitro* validation using EMSA and SPR. As shown in Figure 4, for EMSA, both compounds **3** and **6** inhibited HsrA-DNA binding in a dose-dependent manner, showing weak inhibition at 0.05 mM and stronger inhibition at higher concentrations (0.1–1 mM) (Figure 4A,B). However, the limited solubility of compound **3** in the EMSA reaction buffer prompted us to further examine its binding kinetics and affinity for HsrA using surface plasmon resonance (SPR) analysis.

**Figure 4:**
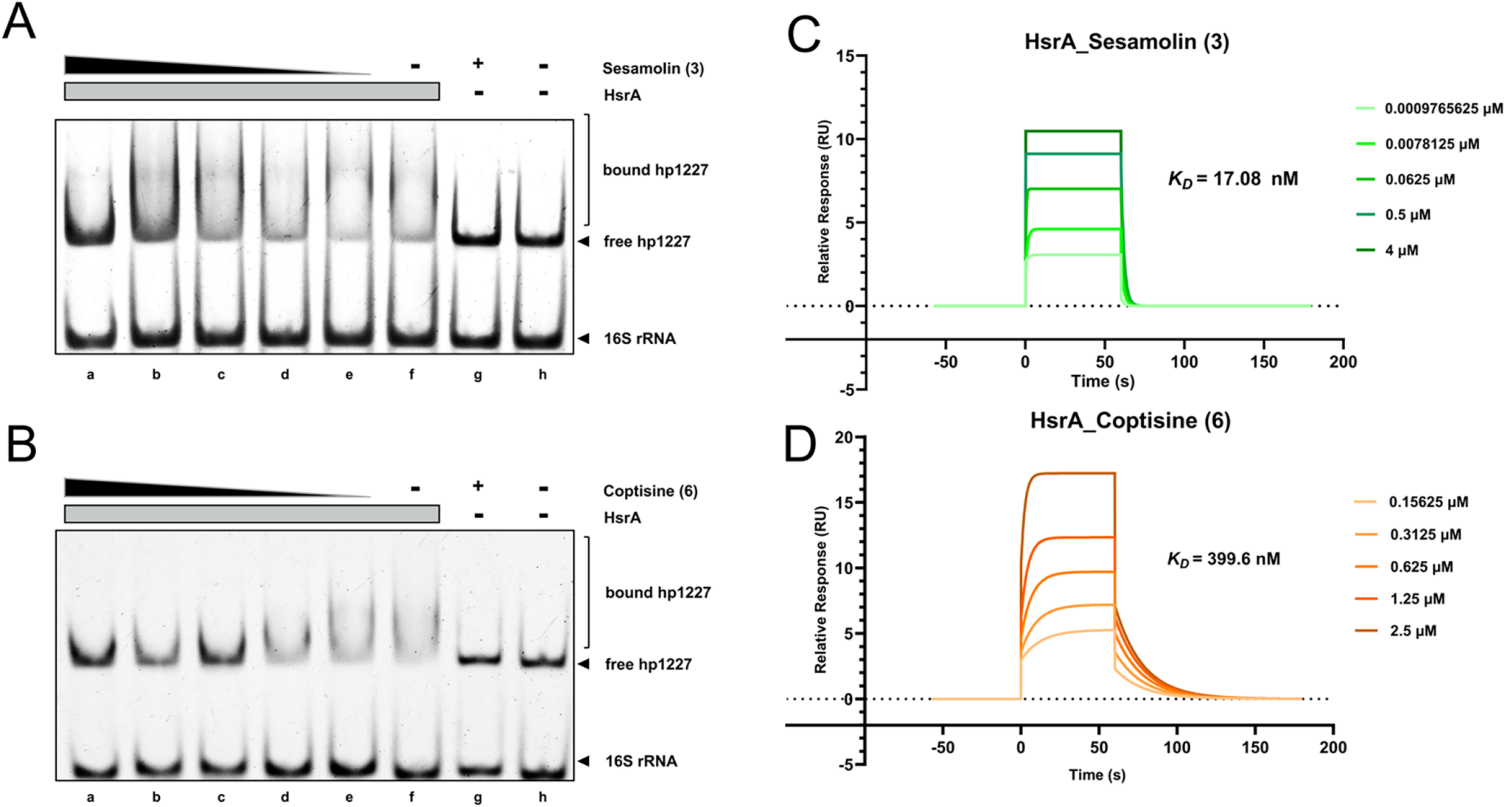
*In vitro* analysis of sesamolin (**3**) and coptisine (**6**) binding to *H. pylori* HsrA. (A, B) EMSA of HsrA-*hp1227* promoter binding inhibition: (A) Compound **3** and (B) compound **6**. Concentration: 0.05–1 mM. (C, D) SPR sensorgrams of HsrA interactions: (C) Compound **3** and (D) compound **6**.

In SPR, HsrA recombinant protein was immobilized on a CM5 sensor with a surface density of over 10,000 response units (RUs). Compound **3** exhibited high binding affinity to HsrA, with a *K*_D_ value of 17.08 nM, χ^2^ (RU^2^) = 0.283 (Figure 4C), whereas compound **6** demonstrated weaker binding affinity, with a *K*_D_ value of 399.6 nM, χ^2^ (RU^2^) = 0.314 (Figure 4D).

### 2.8 Effects of Compounds 3 and 6 on *hp1043* and Associated Genes Expression

RT-qPCR was applied to assess the expression of *hp1043*, HsrA-associated genes (*tlpB* and *flhA*), and other core functional genes (*sodB*, *ureA*, *ureB*, and *secA*) in *H. pylori* following 2 h of exposure to compound **3** (18.9 and 37.8 μM) and compound **6** (49.9 and 99.9 μM). As shown in Figure 5A,B, both compounds downregulated *hp1043* expression in a dose-dependent manner, with compound **3** demonstrating stronger effects at a lower concentration than **6** (37.8 μM *vs* 99.9 μM). For the HsrA-associated gene *tlpB*, compound **3** downregulated its expression at both concentrations, whereas compound **6** upregulated its expression. For *flhA*, both compounds exhibited a dose-dependent effect on its expression but showed opposite regulatory patterns, with compound **3** downregulating and **6** upregulating its expression. The regulatory effects of compounds **3** and **6** on the expression of core functional genes in *H. pylori* were different: *ureA*, *ureB*, and *secA* were downregulated by compound **3** in a dose-dependent manner, whereas this dose-dependent effect was not observed for compound **6**. For *sodB*, both compounds upregulated its expression, but compound **3** exhibited a stronger effect. Overall, compound **3** showed stronger regulatory effects on *hp1043* and its associated genes compared to those of compound **6**.

**Figure 5:**
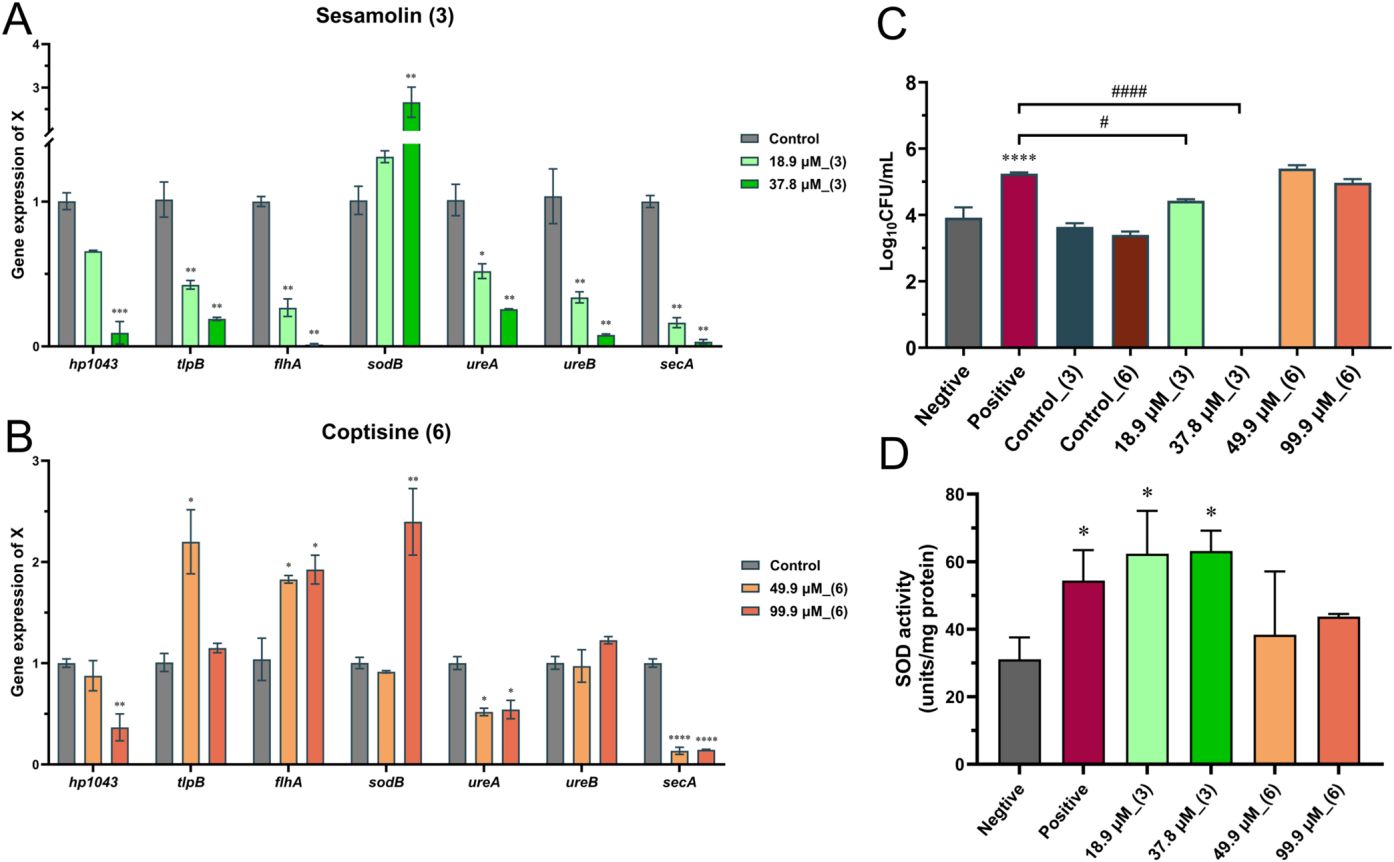
Multimodal analysis of the effects of compounds **3** and **6** on *H. pylori*. (A, B) Effects of compounds **3** and **6** on the expression of *hp1043*, associated genes (*tlpB* and *flhA*), and other core functional genes (*sodB*, *ureA*, *ureB*, and *secA*). The *X*-axis indicates the gene, and the *Y*-axis indicates gene expression levels. *P < 0.05, **P < 0.01, ***P < 0.001, ****P < 0.0001 *vs* control. (C) *H. pylori* urea chemotaxis ability after treatment with compounds **3** and **6**. The *X*-axis indicates different treatment groups, and the *Y*-axis shows log_10_-transformed CFUs in the chemoattractant chamber. ****P < 0.0001 *vs* negative control; ^#^ *P* < 0.05, ^####^ *P* < 0.0001 *vs* positive control. (D) SOD activity in *H. pylori* after treatment with compounds **3** and **6**. The *X*-axis indicates treatment groups, and the *Y*-axis indicates SOD content (units/mg protein). *P < 0.05 *vs* negative control.

### 2.9 Simplified Capillary Chemotaxis Assay

The regulation of compounds **3** and **6** on *tlpB* and *flhA* expression was further confirmed using a simplified capillary chemotaxis assay to assess changes in the urea chemotactic response of *H. pylori*. As shown in Figure 5C, *H. pylori* displayed a strong chemotactic response toward urea in the syringe barrel (positive control *vs* negative control). After treatment with compound **3** at 18.9 μM and 37.8 μM, the chemotactic response of *H. pylori* toward urea was significantly inhibited compared with the positive control, but no significant inhibitory effect was observed with compound **6**.

### 2.10 Determination of Total SOD Activity in *H. pylori*

The regulation of compounds **3** and **6** on *sodB* expression was further confirmed with the SOD assay, since this gene encodes the major superoxide dismutase in *H. pylori*. As shown in Figure 5D, compound **3** significantly increased SOD activity at both 18.9 and 37.8 μM, whereas the effect of compound **6** was much weaker, showing only a slight increase at 49.9 and 99.9 μM.

## 3 Discussion

The rise of antibiotic resistance in *H. pylori* and the side effects associated with current treatments have created an urgent need for screening safer and more effective alternatives, especially those from natural products.^31^ However, the large number of plant species makes traditional experimental screening time-consuming and labor-intensive. To address this gap, we employed an integrated computational–experimental pipeline—combining molecular docking, MD simulations, and *in vitro* evaluation—for the first time in this study and successfully identified sesamolin (**3**) as a potential HsrA inhibitor that has anti-*H. pylori* effects from 13,142 natural compounds in the TCMSP database.

HsrA, an essential and dosage-sensitive transcription factor in *H. pylori*,^23^ is a compelling drug target; however, its indispensability and the limited genetic-manipulation tools available for this bacterium preclude conventional gene-editing or knockout studies, so researchers instead rely on *in vitro* biochemical methods—such as EMSA, SPR, and structure-based compound screening—to probe its function and identify inhibitors.^8,18,22,32^

In this study, HsrA was selected as the virtual screening target because it is an essential, multifunctional, and highly conserved transcriptional regulator in *H. pylori*, making it a promising therapeutic target for *H. pylori* infection.^28^ It has been used in identifying its potential inhibitors in *H. pylori* by using traditional target-based high-throughput screening (HTS). For example, González et al. screened out seven compounds from 1120 FDA-approved drugs,^22^ and repurposing anti-*H. pylori* effects of 1,4-dihydropyridine antihypertensive drugs,^32^ as well as synthesized novel derivatives by targeting HsrA.^33^ Furthermore, a computational methodology coupled with experimental validation was also applied to screen HsrA inhibitors from the ZINC database,^18^ which allowed a rapid reduction of the number of compounds to be tested and reduced the costs of drug discovery. However, to date, there have been no reports of screening natural compounds using this strategy. To our knowledge, this is the first study to screen for HsrA inhibitors using the TCMSP natural-product database from which we assembled an in-house 3D compound library for virtual screening.

The TCMSP database is freely available and includes pharmacochemical data and ADME characteristics of Chinese herbal compounds. In this study, a total of 13,142 natural compounds were retrieved from the TCMSP database and converted into 3D structures to construct an in-house data set for molecular docking. For the initial step of virtual screening, docking simulations were performed in a blind docking manner using these compounds.^34^ The docking score was obtained by using AutoDock Vina. Common rules for drug-like selection (Lipinski’s rule, the Ghose filter rule, Veber’s rule) were used to eliminate compounds without drug-like properties, and 12 compounds with high docking scores and good accessibility were selected by following the method reported by Antoniciello et al.^18^ However, docking results alone cannot provide detailed information on protein–ligand interactions. Therefore, PLIP was used to systematically analyze the interaction patterns of selected compounds (Table S3 and Figure S1), enabling the identification of candidates with favorable binding modes for subsequent MD simulations, as recommended by Salentin et al.^35^

After docking simulations, we first conducted 50 ns MD simulations (termed preliminary simulations in this study) on 12 selected compounds with HsrA, along with MIC and MBC testing, and found that compounds **3** and **6** exhibited the strongest anti-*H. pylori* effects among 12 compounds. Interestingly, although compounds **3** and **6** had similar docking scores and the strongest anti-*H. pylori* activities, their binding stability in MD simulations differed markedly: compound **3** exhibited stable binding characterized by persistent H-bonds (0.571) interactions throughout the simulation, whereas compound **6** performed less well (0.004). To clarify whether this discrepancy was due to insufficient simulation time or incomplete equilibration, we further performed 200 ns MD simulations (termed extended simulations in this study) on compounds **3** and **6**. Consistent with the preliminary MD simulations, the extended simulations still demonstrated that compound **3** maintained persistent H-bonds (0.57), whereas compound **6** showed negligible hydrogen bonding (0), suggesting that compound **3** had greater stability than **6**. Furthermore, in extended simulations, binding free energies of HsrA with compounds **3** and **6** were evaluated by using MM-PBSA and MM-GBSA methods.^36^ The results demonstrated that compound **3** had a lower binding free energy with HsrA compared to compound **6**, indicating that compound **3** binds more stably to HsrA. Furthermore, visualization of residue energy decomposition revealed that the binding sites for compounds **3** and **6** were different: compound **3** binds to the C-terminal DNA-binding site of HsrA (Figure 2D), whereas compound **6** binds to the N-terminal region (Figure 2E). All these results suggest that compound **3** binds more stably to HsrA and specifically at the DNA-binding sites,^18^ indicating that it is more likely to influence HsrA’s regulation of downstream genes by directly affecting its DNA-binding ability. However, the potential impact of this difference needs further investigation.

Because HsrA is an essential gene that cannot be deleted or conditionally silenced in *H. pylori*, *in vivo* genetic approaches are not practical for mapping the regulatory effects of ligand binding.^23^ González et al. therefore used an electrophoretic mobility shift assay (EMSA) to show that small molecules can disrupt the HsrA–DNA complex,^8,22,32^ while Hou et al. demonstrated—using surface plasmon resonance (SPR)—that SPR is well suited for quantifying direct small-molecule/target interactions during postscreen validation.^37^ Based on these findings, we first employed EMSA to test whether the candidate compounds inhibit the binding of HsrA to its cognate DNA, and then used SPR to measure their binding affinities toward HsrA, thereby confirming and characterizing the direct effects of compounds **3** and **6** on this protein. EMSA results demonstrated that both compounds **3** and **6** had inhibitory effects on HsrA–DNA binding, with **6** exhibiting a slightly stronger effect than **3**, which may be attributed to differences in their solubility in aqueous solution.^32^ So in the subsequent SPR tests, 5% DMSO was added to the system to improve the solubility of the small molecules. SPR analysis showed that compound **3** bound to HsrA with a *K*_D_ of 17.08 nM, whereas compound **6** had a *K*_D_ of 399.6 nM (Figure 4), indicating that compound **3** possesses a much higher affinity for HsrA than compound **6**.

As previously reported, HsrA is a DNA-binding activator that autoregulates its own gene and controls core cellular functions in *H. pylori*;^23,30^ inhibitors that impair HsrA-DNA binding are therefore expected to alter the transcription of HsrA-regulated genes. Accordingly, we used RT-qPCR to evaluate changes in HsrA and representative downstream targets, including HsrA-associated genes (*tlpB* and *flhA*) and core functional genes (*ureA*, *ureB*, *sodB*, and *secA*), after 2 h treatments with compounds **3** and **6**, respectively. Consistent with their differential binding to HsrA, compounds **3** and **6** showed distinct transcriptional effects on *hp1043*, HsrA-associated genes, and core functional genes. Both compounds reduced *hp1043* in a dose-dependent manner, but compound **3** achieved stronger suppression at a lower concentration than compound **6**. Compound **3** downregulated *tlpB* at both concentrations and decreased *flhA* in a dose-dependent manner, whereas compound **6** upregulated both *tlpB* and *flhA*. For core functional genes, compound **3** dose-dependently suppressed *ureA*, *ureB*, and *secA*, while compound **6** downregulated only *ureA* without a clear dose response and reduced *secA*. Both compounds upregulated *sodB*, with a stronger effect for compound **3** at lower concentrations. Together, these transcriptional profiles indicate that sesamolin (compound **3**) more effectively disrupts the HsrA regulon, particularly genes linked to chemotaxis, urease function, and protein translocation, which likely contributes to its stronger anti-*H. pylori* activity relative to that of coptisine (compound **6**). In addition, we also evaluated the differences in activity against *H. pylori* between sesamolin and coptisine using time-kill kinetic assays, and found that, at equivalent concentrations, sesamolin exhibited more rapid anti-*H. pylori* activity than coptisine. Besides, in this study, MTZ-resistant strain ATCC 43504 was applied in MIC/MBC evaluation as well as time-kill kinetic assays, since MTZ resistance has the highest resistance rates (up to 79%) in clinical isolates.^38^

Sesamolin (**3**) is a common lignan found in sesame seeds and sesame oil,^39^ possessing favorable drug-like properties, including good lipophilicity (Alog *P* = 2.55), oral bioavailability (OB = 40.13%), and high drug-likeness (DL = 0.88), with excellent antioxidant and anti-inflammatory activities.^40,41^ While sesame oil has been shown to inhibit clinical isolates of *H. pylori* at 25–100% (w/w),^42^ the anti-*H. pylori* activity of sesamolin remains unreported. Here, we demonstrate for the first time that sesamolin (**3**) exhibits potent anti-*H. pylori* activity with MIC and MBC values of 7 and 10 μg/mL, respectively. Notably, sesamolin showed poor aqueous solubility; however, its solubility improved upon addition of DMSO in the SPR assay. Accordingly, future applications should consider solubility-enhancing strategies. Although sesamolin is derived from sesame and animal studies have reported no obvious *in vivo* adverse effects, its cellular cytotoxicity has not been reported and needs to be further studied.^43^

## 4 Conclusions

In this investigation, 12 candidate inhibitors of the essential response regulator HsrA were selected from 13,142 natural products in the TCMSP database through a target-based virtual screening workflow and subsequent antimicrobial evaluation. Among these, sesamolin (**3**) and coptisine (**6**) emerged as leads, with sesamolin (**3**) showing the most promising overall performance in *H. pylori* assays. Sesamolin (**3**) maintained stable binding to HsrA in MD simulations, inhibited HsrA–DNA complex formation in EMSA, and bound HsrA with high affinity by SPR. Consistent with these observations, sesamolin (**3**) produced a faster bactericidal effect than coptisine in time-kill kinetic assays and exerted broader transcriptional modulation of the HsrA regulon, including pathways linked to chemotaxis, urease function, and protein translocation. Collectively, this study identifies sesamolin (**3**) as an HsrA-targeting natural compound and provides a scientific foundation for subsequent studies on mechanism and *in vivo* evaluation, with potential implications for *H. pylori* infection control and prevention.

## 5 Experimental Section

### 5.1 Reagents

Compounds 1–12 were obtained from HerbSubstance Co., Ltd. (Chengdu, China; > 99% purity by HPLC). Dimethyl sulfoxide (DMSO) was purchased from AbMole Bioscience (USA); brain heart infusion (BHI) medium from Hopebio (China) and fetal bovine serum (FBS) were from Bioshark (China). For SPR, recombinant HsrA protein (>95% purity) was obtained from Sangon Biotech (Shanghai, China). CM5 sensor chips and 1× PBS-P+ buffer (20 M phosphate buffer, 2.7 mM KCl, 137 mM NaCl, 0.05% surfactant P20) were obtained from Cytiva (USA). For EMSA, DNA extraction kit (Beyotime, D0063, China); Easy-Load PCR Master Mix (Beyotime, D7255, China); DNA cleanup kit (TianGen, DP214, China); EMSA binding buffer (Beyotime, GS005, China); EMSA loading buffer (Beyotime, GS006, China); TBE (Beyotime, ST187, China); and NA-Red nucleic acid stain (Beyotime, D0128, China) were used. For RT-qPCR, RNA purification kit was obtained from Accurate Biotech Co., Ltd. (Hunan, China). Reverse transcription was carried out with the Evo M-MLV RT Kit (Accurate Biotech Co., Ltd., Hunan, China), and SYBR Green ProTaq HS Premix Kit for qPCR (Accurate Biotech Co., Ltd., Hunan, China). For SOD measurement, the Total Superoxide Dismutase Assay Kit with WST-8 (Beyotime, S0101M, China) was used.

### 5.2 Virtual Screening Preparation

The HsrA structure (PDB ID: 2HQR) was retrieved from the RCSB Protein Data Bank (https://www.rcsb.org/).^44^ The protein structure was prepared by adding Hydrogen atoms using PyMOL (Open-Source v2.5.7).^45^ The evolutionary conservation of the HsrA was assessed using the ConSurf web server (https://consurf.tau.ac.il).^46,47^ The potential drug-binding sites of the protein were identified using the DoGSiteScorer tool in ProteinsPlus (https://proteins.plus).^48^ All molecules of natural compounds were downloaded in MOL2 format from the TCMSP database (https://www.tcmsp-e.com/) and converted to 3D coordinates using Open Babel GUI (v3.1.1)^49^ to construct an in-house data set for the following virtual screening.

### 5.3 Molecular Docking and Molecular Dynamics (MD) Simulations

Molecular docking, downloaded natural compounds with HsrA, was performed using AutoDock Vina v1.1.2 with parameter optimization in AutoDock Tools and Vina v1.5.7.^50–52^ A blind docking strategy was implemented with a customized docking box (center: *x* = 2.278, *y* = 16.528, *z* = −7.083; dimensions: 118 Å × 112 Å × 88 Å). The top-ranked docked pose for each ligand was retained, and the compounds were subsequently filtered by Lipinski’s rule of five, the Ghose filter rule, and Veber’s rule.^53–55^ Furthermore, the Protein–Ligand Interaction Profiler (PLIP) online platform (https://plip-tool.biotec.tu-dresden.de) was used to analyze the interactions with HsrA of selected compounds with strong anti-*H. pylori* activity, followed by MD simulation.^56^

The preliminary (50 ns) and extended (200 ns) MD simulations were performed using GROMACS 5.0.7 ^57^ with the AMBER 14 SB force field and the TIP3P water model.^58,59^ Ligand parameters were assigned *via* Sobtop 1.0 (GAFF/UFF).^60^ A 12 Å buffer zone between the complex and simulation box boundary was maintained to avoid edge effects.^61^ The system was solvated in 0.15 M NaCl, energy-minimized, equilibrated under NVT and NPT ensembles (310 K, 1 atm),^61^ and simulated using the V-rescale thermostat, with trajectory analyses (RMSD, RMSF, H-bonds, and distances) performed using GROMACS tools and visualized with PyMOL. Pocket occupancy (%) was calculated in GROMACS (gmx select) as the fraction of trajectory frames with ≥1 ligand heavy atom within 4.0 Å of any atom of any residue assigned to a given DoGSiteScorer-defined pocket.^35^ H-bond interactions were defined by geometric thresholds (donor–acceptor distance <3.5 Å; D–H–A angle >120°). After MD simulation, binding free energies were computed from the stable conformations in 200 ns trajectories using MM-GBSA and MM-PBSA,^62,63^ with energy convergence validated by <3.6 kcal mol^–1^ fluctuation thresholds.^64^

### 5.4 *H. pylori* Strains and Culture Conditions

The MTZ-resistant reference *H. pylori* strain ATCC 43504 was used for antimicrobial evaluation. It was cultured in BHI broth (10% FBS) and Columbia blood agar (CBA) plates (7% sterile defibrinated sheep blood) under microaerobic conditions (85% N_2_, 10% CO_2_, and 5% O_2_) at 37 °C.

### 5.5 Evaluation of the Anti-*H. pylori* Activity of Natural Compounds *In Vitro*

The antimicrobial activity of natural compounds against *H. pylori* was evaluated by determining the MIC and MBC using the microdilution broth method. For MIC and MBC assays, serial dilutions of compounds (1–1024 μg/mL) were mixed with *H. pylori* inoculum (1 × 10^6^ CFU/mL) in 96-well plates. After 48 h of culture, MIC values were recorded as the lowest concentration showing no visible bacterial growth. MBC was determined by subculturing 10 μL from clear wells onto CBA and defined as the lowest concentration achieving ≥99.9% bacterial lethality after 48 h. Time-kill kinetics assays were conducted using compound concentrations of 20, 40, 60, and 80 μM. At specified time points (0, 2, 4, 8, 12, 24, and 48 h post-treatment), culturing aliquots were collected, followed by serially diluting in sterile normal saline (NS), and plated on CBA plates for viable colony counting. All antimicrobial experiments included three technical replicates per condition, and the entire experiment was independently repeated twice.

### 5.6 Electrophoretic Mobility Shift Assay (EMSA)

The EMSA was performed using the *hp1227* probe (a known HsrA-binding sequence) and a nonspecific 16S rRNA control probe; both probes were obtained by PCR amplification from *H. pylori* ATCC 43504 genomic DNA.^30^ PCR products were verified by using 1.5% agarose gel electrophoresis, purified, and diluted to 10 ng/μL. The binding reaction mixture (25 μL final volume) contained 50 ng of *hp1227* probe, 50 ng of 16S rRNA competitor DNA, 4 μM purified HsrA, and tested compounds at graded concentrations (0.05, 0.1, 0.2, 0.5, 1 mM). Purified HsrA (final concentration: at 4 μM), *hp1227*/16S rRNA probe (50 ng for each), and tested compounds at graded concentrations (0.05, 0.1, 0.2, 0.5, 1 mM) were added to the EMSA binding buffer (final volume: at 25 μL), respectively. After mixing, the samples were incubated at 25 °C for 30 min and then resolved on 5% native polyacrylamide gels (0.5× TBE buffer, 90 V, 75 min), stained with a fluorescent nucleic acid dye (1:1000 dilution), and imaged using an iBright FL1500 system.

### 5.7 Surface Plasmon Resonance (SPR)

Real-time interactions between HsrA and test compounds were analyzed by using SPR with Biacore T200 (GE Healthcare) at 25 °C. In brief, HsrA was covalently immobilized on a CM5 sensor chip using a pH = 4.0 acetic acid solution. Compounds, prepared at optimized concentrations, were injected over the chip surface for 60 s at a flow rate of 30 μL/min, and binding responses (resonance units, RU) were recorded. PBS-P+ with 5% DMSO was used as a running buffer to enhance compound solubility and correct for solvent effects. Data acquisition and analysis were performed by using Biacore T200 Control and Evaluation Software. Equilibrium dissociation constants (*K*_D_) were calculated using a 1:1 binding model.

### 5.8 Quantitative Reverse Transcription Polymerase Chain Reaction (RT-qPCR)

The effects of natural compounds on the expression of *hp1043* and related genes in *H. pylori* were evaluated by quantitative PCR (RT-PCR). Selected compounds were incubated with *H. pylori* strains (ATCC 43504) under microaerophilic conditions (85% N_2_, 10% CO_2_, 5% O_2_) at 37 °C for 2 h. The bacteria pellets were obtained by centrifugation, and their total RNA was purified, followed by reverse transcription using 500 ng of RNA. SYBR Green-based qPCR was performed in a 20 μL reaction with gene-specific primers (Table S4) under the following cycling conditions: 95 °C for 30 s, followed by 40 cycles of 95 °C for 5 s and 60 °C for 30 s. Relative gene expression was calculated by using the 2^–ΔΔCt^ method with *H. pylori* 16S rRNA as the endogenous reference. Experiments were conducted in triplicate, each with three technical replicates.

### 5.9 Simplified Capillary Chemotaxis Assay

Simplified capillary chemotaxis assays were conducted according to the method reported by Mazumder et al., with modifications.^65^ Briefly, a 200 μL pipet tip was used as a chamber to hold the *H. pylori* suspension (OD_600_ = 0.1) together with the tested compounds. The needle of a 1 mL disposable syringe served as the chemotaxis capillary, while the syringe barrel was filled with 10 mM urea solution, a classical chemoattractant for *H. pylori*. The syringe with attached needle was then inserted into the pipet tip chamber containing the bacterial suspension, and the assembled apparatus (Figure 6) was incubated for 30 min under microaerophilic conditions (85% N_2_, 10% CO_2_, 5% O_2_). After incubation, the effect of the selected compounds on *H. pylori* chemotaxis was evaluated by quantifying the number of bacteria that migrated into the urea solution in the syringe barrel, which was then serially diluted in NS and plated on CBA plates for viable colony counting. As shown in Table 5, the negative control group contained only *H. pylori* in the pipet tip and NS in the syringe barrel. In the positive control group, *H. pylori* were present in the pipet tip, with 10 mM urea added to the syringe barrel. The compound control group consisted of *H. pylori* and either compound **3** or **6** in the pipet tip, with NS in the syringe barrel. In the treatment groups, *H. pylori* and either compound **3** (18.9 μM or 37.8 μM) or compound **6** (49.9 μM or 99.9 μM) were included in the pipet tip, with 10 mM urea added to the syringe barrel.

**Figure 6:**
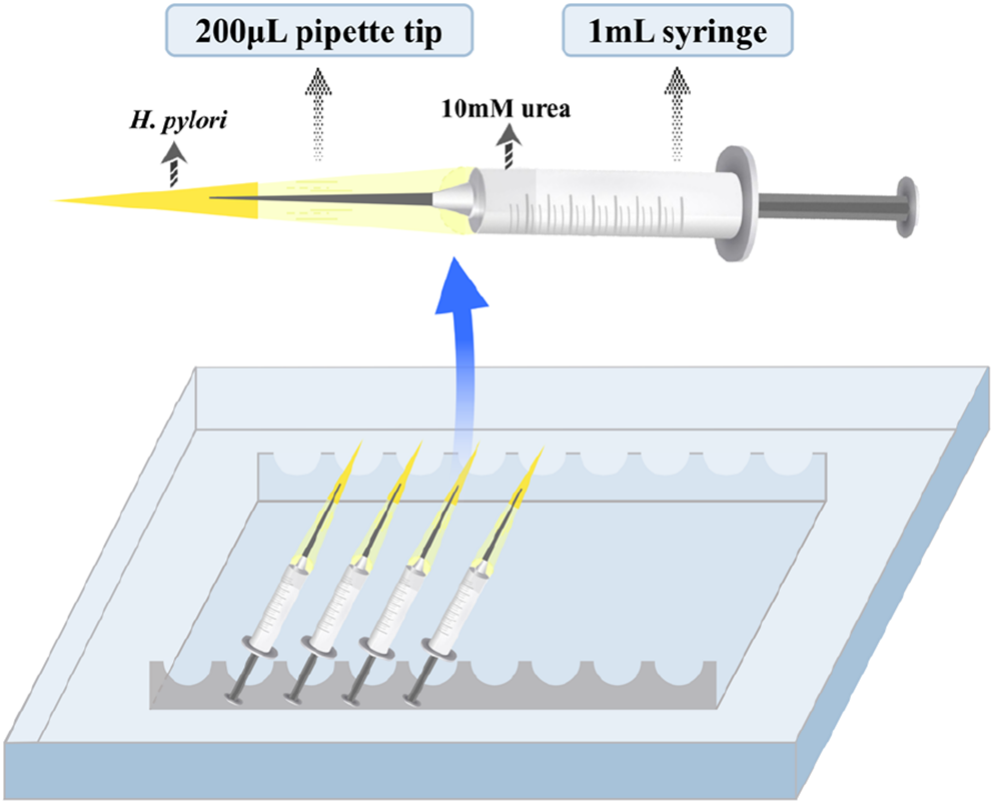
Diagram of the assembled simplified capillary apparatus.

**Table 5:** Experimental Group Setup of Simplified Capillary Assay

group	pipet tip	syringe barrel
negative control	*H. pylori*	NS
positive control	*H. pylori*	10 mM urea
compound control	*H. pylori* + compound **3** or **6**	NS
treatment groups	*H. pylori* + compound **3** or **6**	10 mM urea
	*H. pylori* + compound **3** or **6**	10 mM urea

### 5.10 Determination of Total SOD activity

The influence of the tested compounds on the total SOD activity of *H. pylori* was measured using an SOD assay kit (Beyotime, S0101M). Briefly, the SOD activity of five groups: negative control (untreated), positive control (64 μg/mL MTZ), and treatment groups (tested compounds at 1 × MIC and 2 × MIC concentrations) were incubated at microaerophilic conditions for 2 h, followed by centrifugation. The obtained pellets were lysed and subjected to the WST-8 assay according to the manufacturer’s protocol. Absorbance was measured at 450 nm, with 600 nm as the reference. SOD activity was calculated and expressed as units per mg protein, with one unit defined as the amount of enzyme required to inhibit the xanthine oxidase system by 50%.

### 5.11 Statistical Analysis

Data obtained from MD simulations were expressed as the mean ± standard deviation (SD) to indicate variability. All statistical analyses were performed using GraphPad Prism software (version 10.3.1; GraphPad Inc., San Diego, CA, USA). For comparisons of gene expression levels among experimental groups in RT-qPCR experiments, we applied one-way analysis of variance (ANOVA) followed by Tukey’s post hoc test for multiple comparisons. Statistical significance was defined as *P* < 0.05 for all analyses.

## Supplementary Material





## Notes

Natural compounds (docking scores ≤ −7.0 kcal mol^–1^) and their drug-likeness (Table S1); twelve selected natural compounds with 2D structures, molecular formulas, and sources (Table S2); ligand-binding pocket descriptors of HsrA; (Table S3); binding energy and interaction profiles analyzed by PLIP (Table S4); pocket occupancy percentages of compounds in MD simulations (50/200 ns, Table S5); and primer sequences used in this study (Table S6) (XLSX)
Molecular interaction diagrams (Figure S1); RMSD (Å) of the complexes, HsrA backbone, and ligands during 50/200 ns MD simulations (Figures S2 and S4); and interchain distances of chain A-B COM; NTD-NTD and DBD-DBD (Cα-COG) during 50/200 ns MD simulations (Figures S3 and S5) (PDF)
