# Supplementary material for: Discovery of Sesamolin as a Potential Anti-Helicobacter pylori Agent by Virtual Screening against the Essential Response Regulator HsrA with In Vitro Evaluation
Source: ACS Omega. 2025 Nov 11;10(46):56458–70. doi: 10.1021/acsomega.5c08531 (PMC12658632; doi:10.1021/acsomega.5c08531)
Supplement: Supplementary file 2 [file ao5c08531_si_002.pdf]

Discovery of sesamolin as a potential anti-*Helicobacter pylori* agent by virtual screening against the essential response regulator HsrA with *in vitro* evaluation

Yihang Xu[1], *Shuhan Luo*[1], *Fen Yang*[1], *Baochao Zhang*[1], *Xiaoqin He*[1], *Lili Liao*[1],

Juan Liao[1], *Tianli Zheng*[1*] *and Xiaofang Pei*[1*]

[1] West China School of Public Health and West China Fourth Hospital, Sichuan University, Chengdu, 610041, China

[*]Corresponding Author:

Xiaofang Pei. Email: xxpei@scu.edu.cn

Tianli Zheng. Email: 1125338361@qq.com

Table of Contents

Page 1. Figure S1 Molecular interaction analysis of natural compounds with the HsrA.

Page 2. Figure S2 RMSD (Å) of the complexes, HsrA backbone and ligands during 50 ns MD simulations for twelve natural compounds.

Page 3. Figure S3 Interchain distances (Å): chain A-B COM; NTD-NTD and DBD-DBD (Cα-COG) during 50 ns MD simulations for the twelve natural compounds.

Page 4. Figure S4 RMSD (Å) of the complexes, HsrA backbone and ligands during 200 ns MD simulations for (A) Sesamolin (**3**). (B) Coptisine (**6**).

Page 4. Figure S5 Interchain distances (Å): chain A-B COM; NTD-NTD and DBD-DBD (Cα-COG) during 200 ns MD simulations for (A) Sesamolin (**3**). (B) Coptisine (**6**).

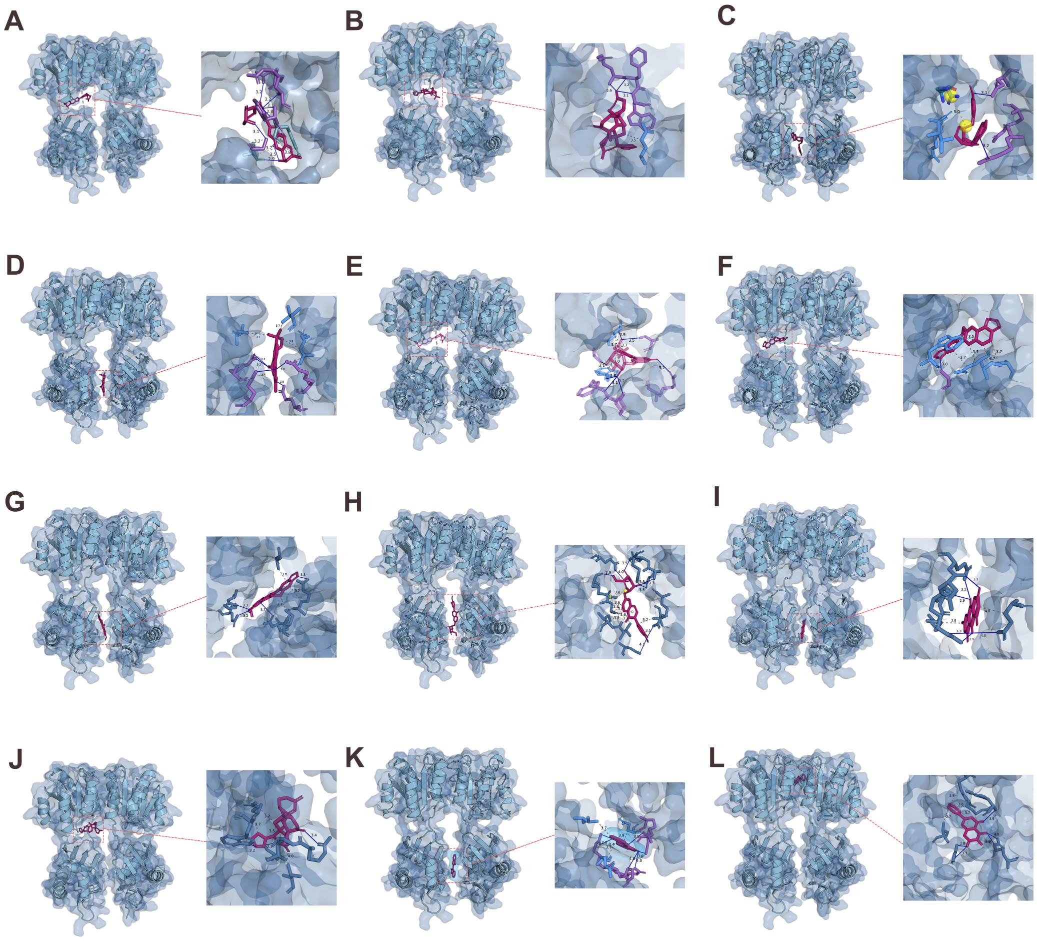

Figure S1 Molecular Interaction Analysis of Natural Compounds with the HsrA: The HsrA is shown in blue, with compounds depicted in maroon. This Figure illustrates the specific docking sites for each compound within the HsrA structure. (A) Mulberroside C (**1**). (B) Limonin (**2**). (C) Sesamolin (**3**). (D) Tanshinone IIA (**4**). (E) Glycitin (**5**). (F) Coptisine (**6**). (G) Berlambine (**7**). (H) Calycosin-7-O-beta-D-glucopyranoside (**8**). (I) Emodin (**9**). (J) Obacunone (**10**). (K) Indirubin (**11**). (L) Baicalein (**12**). Light blue sticks indicate residues involved in hydrophobic interactions, purple for H-bonds, and yellow spheres for salt bridges. Interaction forces are depicted by solid blue lines for H-bonds, grey dashed lines for hydrophobic interactions, and yellow dashed lines for salt bridges.

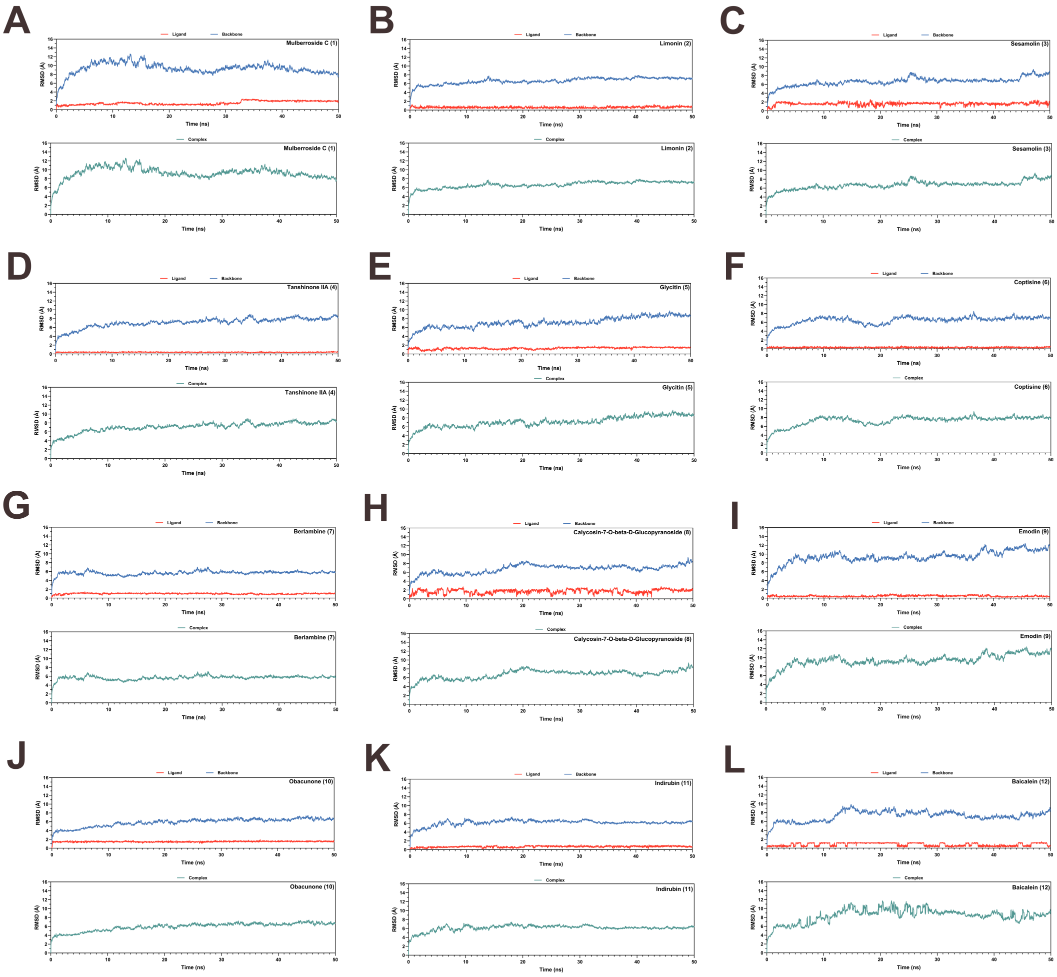

Figure S2 RMSD (Å) of the complexes, HsrA backbone and ligands during 50 ns MD simulations for twelve natural compounds. (A) Mulberroside C (**1**). (B) Limonin (**2**). (C) Sesamolin (**3**). (D) Tanshinone IIA (**4**). (E) Glycitin (**5**). (F) Coptisine (**6**). (G) Berlambine (**7**). (H) Calycosin-7-O-beta-D-glucopyranoside (**8**). (I) Emodin (**9**). (J) Obacunone (**10**). (K) Indirubin (**11**). (L) Baicalein (**12**).

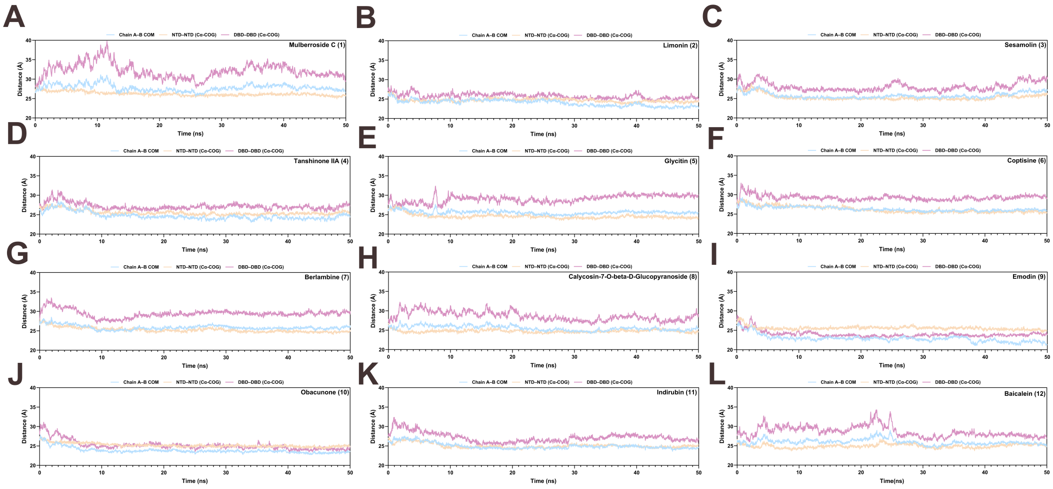

Figure S3 Interchain distances (Å): chain A-B COM; NTD-NTD and DBD-DBD (Cα-COG) during 50 ns MD simulations for the twelve natural compounds. (A) Mulberroside C (**1**). (B) Limonin (**2**). (C) Sesamolin (**3**). (D) Tanshinone IIA (**4**). (E) Glycitin (**5**). (F) Coptisine (**6**). (G) Berlambine (**7**). (H) Calycosin-7-O-beta-D-glucopyranoside (**8**). (I) Emodin (**9**). (J) Obacunone (**10**). (K) Indirubin (**11**). (L) Baicalein (**12**).

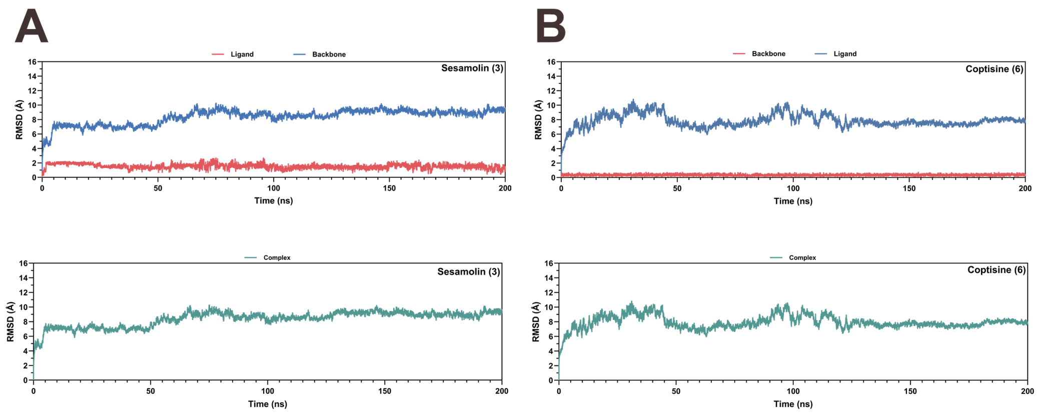

Figure S4 RMSD (Å) of the complexes, HsrA backbone and ligands during 200 ns MD simulations for (A) Sesamolin (**3**). (B) Coptisine (**6**).

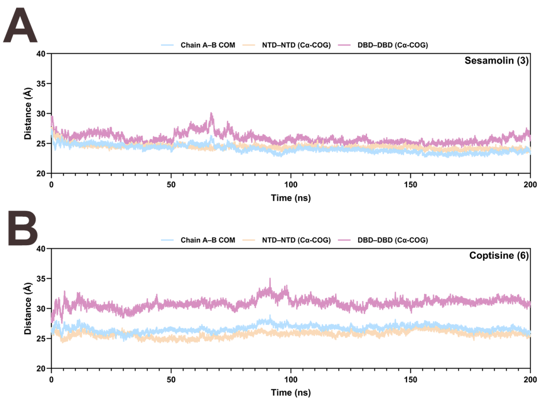

Figure S5 Interchain distances (Å): chain A-B COM; NTD-NTD and DBD-DBD (Cα-COG) during 200 ns MD simulations for (A) Sesamolin (**3**). (B) Coptisine (**6**
